# Characterization of silicon pore optics for the NewAthena X-ray observatory in the PTB laboratory at BESSY II

**DOI:** 10.1107/S1600577524004090

**Published:** 2024-06-24

**Authors:** M. Krumrey, D. Skroblin, L. Cibik, M. Collon, G. Vacanti, N. Barrière, E. Hauser, M. Bavdaz

**Affiliations:** a Physikalisch-Technische Bundesanstalt (PTB) Abbestrasse 2-12 10587Berlin Germany; b cosine Research BV, Warmonderweg 14, 2171 AHSassenheim, The Netherlands; c ESTEC Keplerlaan 1 2200 AGNoordwijk The Netherlands; DESY, Germany

**Keywords:** silicon pore optics, optics characterization, X-ray reflectance, X-ray optics characterization

## Abstract

The New Advanced Telescope for High ENergy Astrophysics (NewAthena) will be the largest space-based X-ray observatory ever built. It will have an effective area above 1.1 m^2^ at 1 keV, which corresponds to a polished mirror surface of about 300 m^2^ due to the grazing incidence. As such a mirror area is not achievable with an acceptable mass even with nested shells, silicon pore optics (SPO) technology will be utilized. In the PTB laboratory at BESSY II, two dedicated beamlines are in use for their characterization with monochromatic radiation at 1 keV and a low divergence well below 2 arcsec: the X-ray Pencil Beam Facility (XPBF 1) and the X-ray Parallel Beam Facility (XPBF 2.0), where beam sizes up to 8 mm × 8 mm are available while maintaining low beam divergence. This beamline is used for characterizing mirror stacks and controlling the focusing properties of mirror modules (MMs) – consisting of four mirror stacks – during their assembly at the beamline. A movable CCD based camera system 12 m from the MM registers the direct and the reflected beams. The positioning of the detector is verified by a laser tracker. The energy-dependent reflectance in double reflection through the pores of an MM with an Ir coating was measured at the PTB four-crystal monochromator beamline in the photon energy range 1.75 keV to 10 keV, revealing the effects of the Ir *M* edges. The measured reflectance properties are in agreement with the design values to achieve the envisaged effective area.

## Abstract 2

Mirror modules for the optics of the X-ray observatory NewAthena are assembled and characterized at dedicated synchrotron radiation beamlines. The reflectance of a fully assembled and coated module was determined in a wide energy range.

## 1. Introduction

X-ray observatories in space are required to answer fundamental questions in astrophysics related to, for example, the growth of black holes and the formation of the large-scale structure of the universe. Compared with the X-ray observatories in use today like Chandra from NASA (Weisskopf *et al.*, 2002 ▸) and XMM Newton from ESA (Jansen *et al.*, 2001 ▸), which were both launched in 1999, a new observatory should have a larger effective area, higher spectral and angular resolution, and a larger field of view. The spectral resolution is mainly dependent on the detector, while effective area and angular resolution depend on the X-ray optics. As X-rays are only reflected for very shallow incidence angles, an effective area of 1 m^2^ corresponds to a real mirror surface of about 300 m^2^. To achieve such a large area in space with lightweight, stiff optics with high surface quality, silicon pore optics (SPO) were developed. Several possible observatories based on SPOs like XEUS (Beijersbergen *et al.*, 2004 ▸) and IXO (Collon *et al.*, 2009 ▸) were investigated by ESA before selecting Athena, the Advanced Telescope for High Energy Astrophysics, in 2014. Due to cost constraints, the mission was re-formulated in 2023 and is now called NewAthena (Bavdaz *et al.*, 2023 ▸). The focal length is still 12 m, the angular resolution will be <9 arcsec and the effective area at 1 keV will be >1.1 m^2^. To achieve this area, about 500 mirror modules (MMs) are required.

To produce the mirrors, commercial 300 mm double-sided super-polished silicon wafers are diced, ribbed and wedged to create rectangular plates with ribs on the bottom side (Collon *et al.*, 2022 ▸). Mirror stacks with up to 38 plates are assembled by a stacking robot at the Dutch company cosine so that pores of about 1 mm × 1 mm are formed. Prior to stacking, the reflecting surface is coated with Ir to enhance the reflectance at higher photon energies up to about 10 keV. As the plates are directly bonded to each other, only stripes on the reflecting surface between the ribs (on the opposite side of the ribs) are coated.

**Figure 1:**
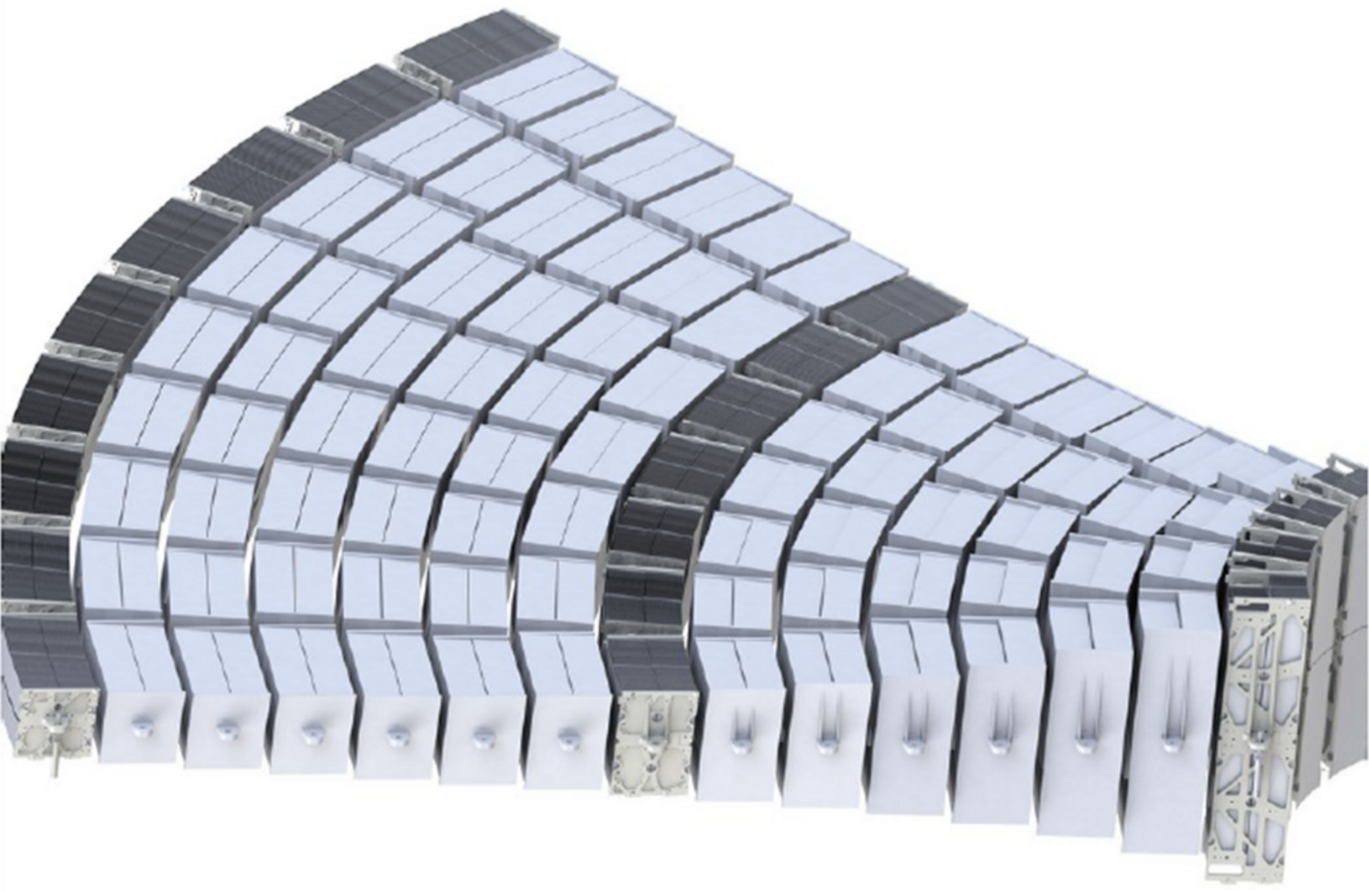
Section of the X-ray lens for Athena, composed of MMs grouped in 15 rows depending on the radius.

An X-ray optical unit (XOU) with double reflection can be described as modified Wolter 1 geometry, where the primary stack is conical and the secondary has a circular meridional curvature. An MM is comprised of two XOUs. Several hundred MMs are required to form a large circular X-ray lens with a diameter of about 2.5 m. The MMs will be grouped in 13 or 15 rows, depending on the radius, thus the distance from the optical axis. A section of the lens is shown in Fig. 1 ▸ (Ferreira *et al.*, 2023 ▸). The geometry of the MM varies depending on the row. The modules are smallest and longest for the innermost row with a very shallow incidence angle, and they are widest and shortest for the outermost row with incidence angles up to 1.4°. Additionally, every plate within the stack is wedged by about 0.001° so that all incoming photons arrive at a common focus.

In this paper, the dedicated beamlines XPBF 1 and XPBF 2.0 for the characterization and assembly of MMs in the PTB laboratory at BESSY II are described as well as the energy-dependent reflectance measurements of an Ir-coated MM in the spectral range from 1.75 keV to 10 keV. X-ray fluorescence measurements on the top surface are also presented. An outlook is given for the mass-production of MMs at additional beamlines and future measurements.

## 2. PTB laboratory at BESSY II and dedicated beamlines for the characterization of SPOs

BESSY II is a third-generation electron storage ring operated by the Helmholtz–Zentrum Berlin (HZB) in Berlin, Germany, with an electron energy of 1.7 GeV and a characteristic photon energy at the bending magnets of 2.5 keV. The Physikalisch–Technische Bundesanstalt (PTB), Germany’s national metrology institute, has established a laboratory for metrology with synchrotron radiation already in 1998 (Beckhoff *et al.*, 2009 ▸). Here, eight beamline branches can be used simultaneously; two of them are dedicated beamlines financed by ESA for the characterization of SPOs.

The X-ray Pencil Beam Facility (XPBF 1) has been in operation since 2005. Two apertures at distances of 12.2 m and 30.5 m from the dipole source form a pencil beam with a typical diameter of 100 µm and a divergence below 1 arcsec. An Si(111) channel-cut monochromator was initially used to select a photon energy of 2.8 keV. However, it was later replaced by a pair of W/B_4_C multilayers to reduce the photon energy to 1 keV. The SPO under investigation is placed in a vacuum chamber on a hexapod, with angular positioning controlled by two electronic autocollimators to below 1 arcsec. The reflected beam is registered at a distance 5 m from the SPO with a CCD-based camera system using a phosphor-coated vacuum window and tandem optics for the visible light (Krumrey *et al.*, 2010 ▸).

**Figure 2:**
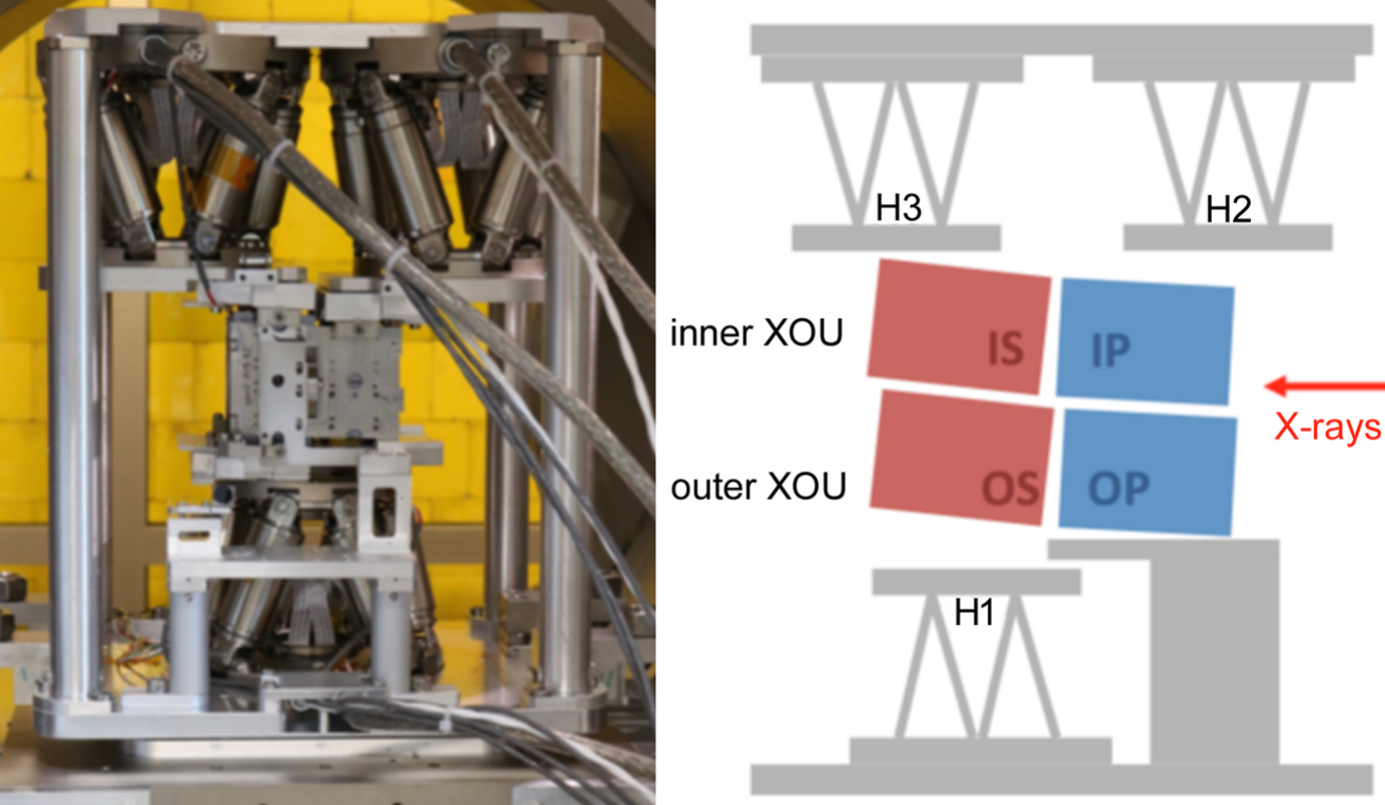
MM assembly at XPBF 2.0 using three small hexapods to align the mirror stacks: OS (outer secondary), IP (inner primary) and IS (inner secondary) with respect to OP (outer primary). For the alignment with X-rays, the setup is placed on the main hexapod in the vacuum sample chamber.

**Figure 3:**
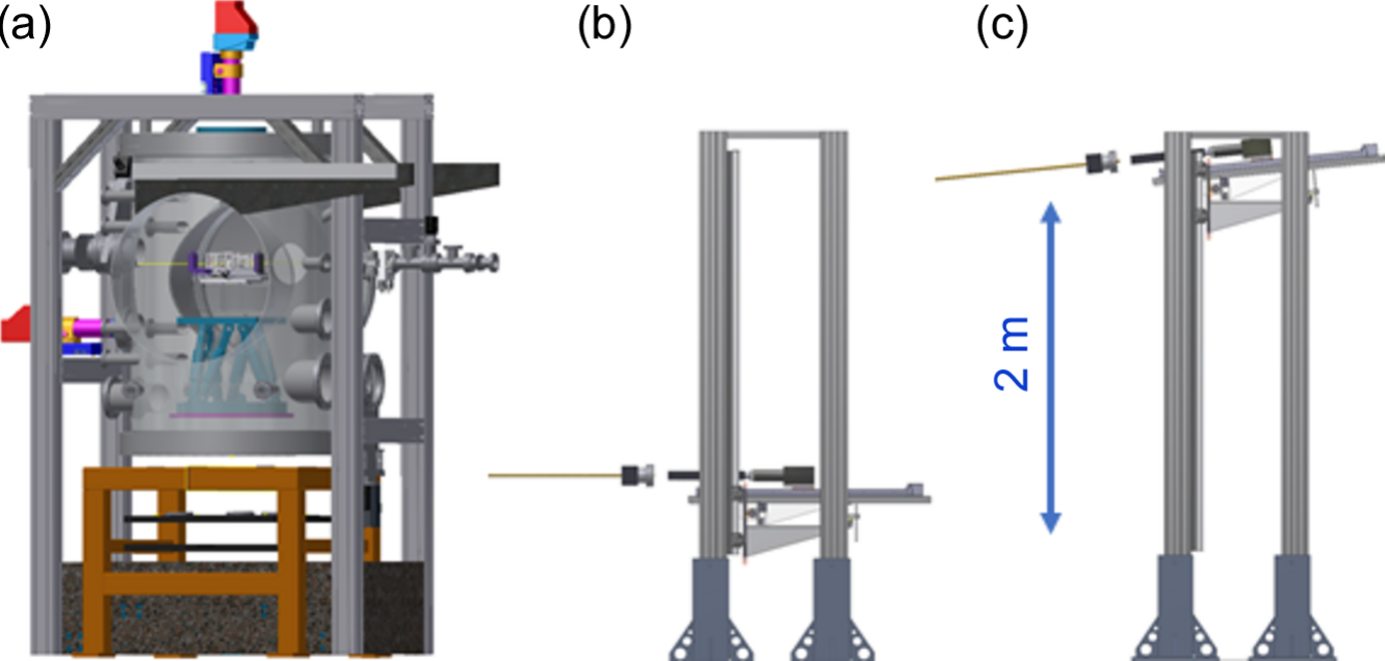
(*a*) Drawing of the vacuum sample chamber at XPBF 2.0 with the main hexapod and two electronic autocollimators and CCD-based detector system on the vertical translation stage at a distance of 12 m from the chamber in the position for the (*b*) direct beam and (*c*) the reflected beam.

The X-ray parallel beam facility XPBF 2.0 was inaugurated in 2016 (Krumrey *et al.*, 2016 ▸). The low divergence is achieved using a toroidal mirror instead of pinholes, which allows for varying the beam size. This enables the illumination of large areas of the SPO while keeping the beam divergence below 2 arcsec. As the toroidal mirror is coated with a W/Si multilayer, it also acts as monochromator for the same energy of 1 keV. Compared with XPBF 1, the sample chamber is bigger and the two larger doors allow the installation of not only an MM for characterization but a setup consisting of three smaller hexapods on the main hexapod to assemble an MM by aligning three mirror stacks with respect to the first stack, as shown in Fig. 2 ▸ (Barrière *et al.*, 2021 ▸). The angular positioning of the main hexapod is controlled with two electronic autocollimators [Fig. 3 ▸(*a*)] to guarantee an angular positioning accuracy of 0.7 arcsec. The alignment and quality of the MM can be controlled by verifying the focusing properties with a CCD-based detector system at a distance of 12 m, which corresponds to the focal length of NewAthena. To capture both the direct and reflected beams, even at steep incidence angles, the detector system has a tilt mechanism and a vertical travel range of 2.1 m as shown in Figs. 3 ▸(*b*) and 3 ▸(*c*). Additionally, the detector can be translated horizontally and by 1 m in the beam direction. As the position of the reflected beam must be known with high accuracy, the location of the detector is monitored with a laser tracker (Handick *et al.*, 2020 ▸). The main parameters of both beamlines are summarized in Table 1 ▸.

## 3. Reflectance measurements at the FCM beamline

**Figure 4:**
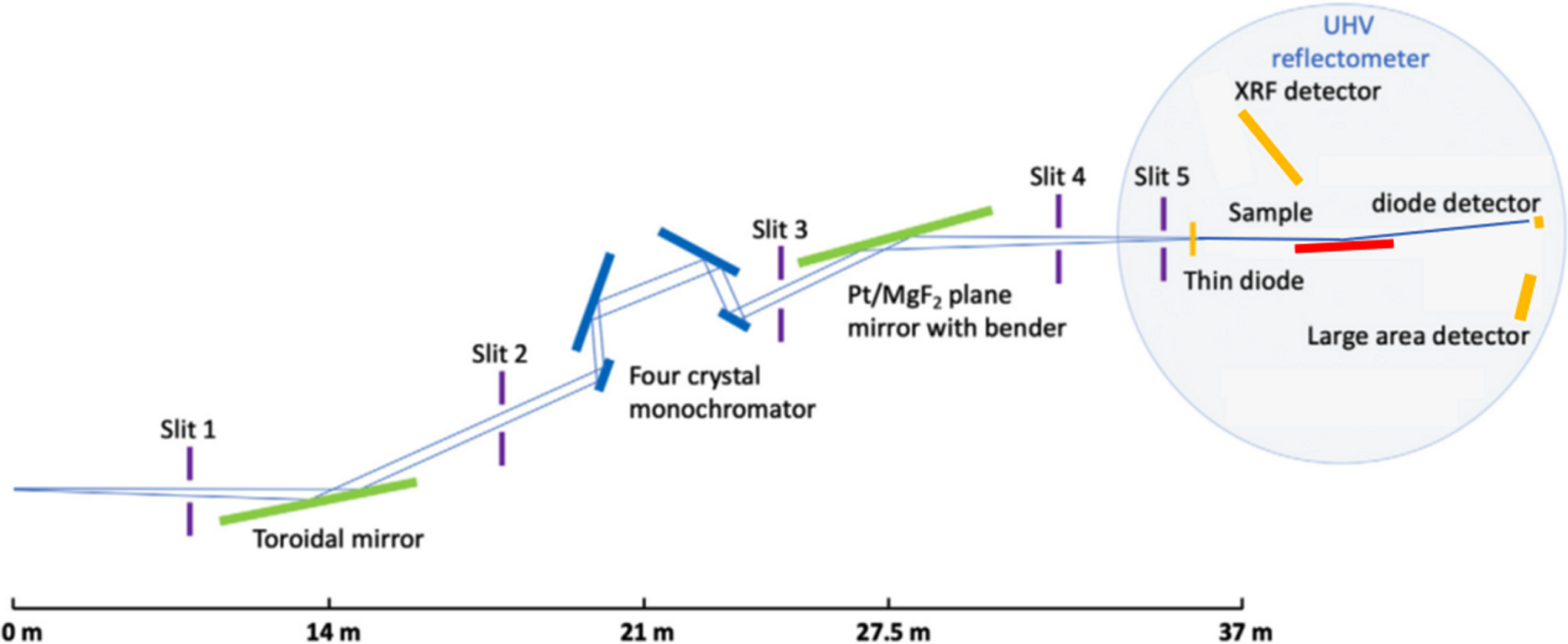
Layout of the FCM beamline in the PTB laboratory at BESSY II with the attached UHV reflectometer.

For photon energy-dependent reflectance measurements of NewAthena mirror coatings, other beamlines in the PTB laboratory are used, especially the four-crystal monochromator (FCM) beamline shown in Fig. 4 ▸, where the energy range from 1.75 keV to 10 keV can be covered by using sets of InSb(111) and Si(111) crystals that can be interchanged under vacuum (Krumrey *et al.*, 1998 ▸). A Pt-coated toroidal mirror with a grazing incidence angle of 0.43° is used to focus the beam horizontally and collimate it vertically. Behind the FCM, a plane mirror with a bender can be used for vertical focusing. To keep the higher-order power contributions below 10^−3^ throughout the energy range, this mirror has an MgF_2_ and a Pt coating stripe (Krumrey & Ulm, 2001 ▸). The attached UHV reflectometer with a load-lock features horizontal and vertical sample travel ranges of 160 mm and 20 mm, respectively (Fuchs *et al.*, 1995 ▸). It is equipped with Si and GaP photodiodes (with and without a slit) and a large-area PILATUS 100k hybrid pixel detector at a distance of about 200 mm on the rotatable detector arm (Skroblin *et al.*, 2020 ▸). In addition, an energy-dispersive silicon drift detector (SDD) at 45° with respect to the incoming beam is available for X-ray fluorescence (XRF) measurements.

In cooperation with cosine and the Danish Technical University (DTU), various material combinations on test samples have been investigated, including their reflectance stability over time and after cleaning procedures required for mirror stacking (Jafari *et al.*, 2020 ▸). Recently, the reflectance improvement by trilayers compared with bilayers has been studied (Windt *et al.*, 2023 ▸), as well as the X-ray transmittance of optical blocking filters, which are also required for high-energy space missions such as NewAthena (Sciortino *et al.*, 2024 ▸). The first characterization of a fully assembled MM with double reflection in the entire energy range of the FCM beamline is reported in this paper.

**Figure 5:**
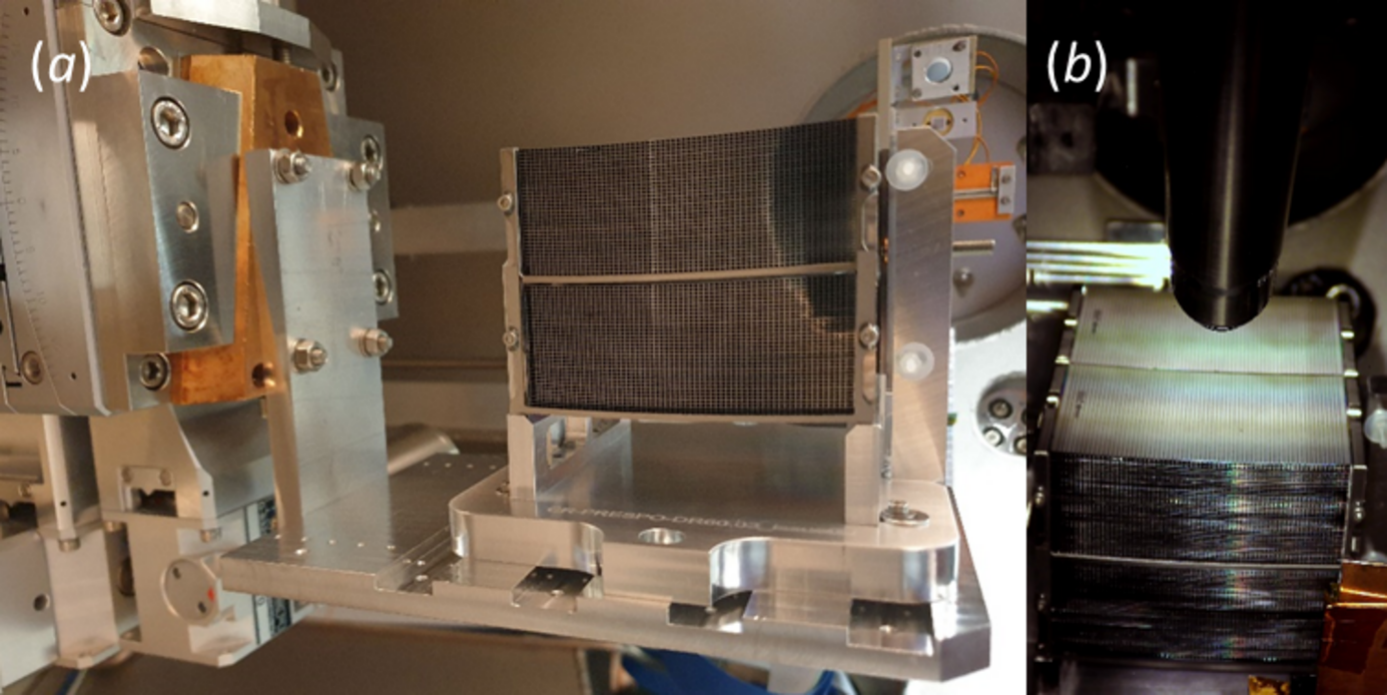
(*a*) Fully assembled MM in the reflectometer. In the beam direction, the pores of the MM can be seen as well as the goniometer head for alignment on the left and the semiconductor photodiodes for the reflectance measurements in the back. (*b*) From a top view, the coating stripes on the top surface and the SDD for the XRF detection are visible.

**Figure 6:**
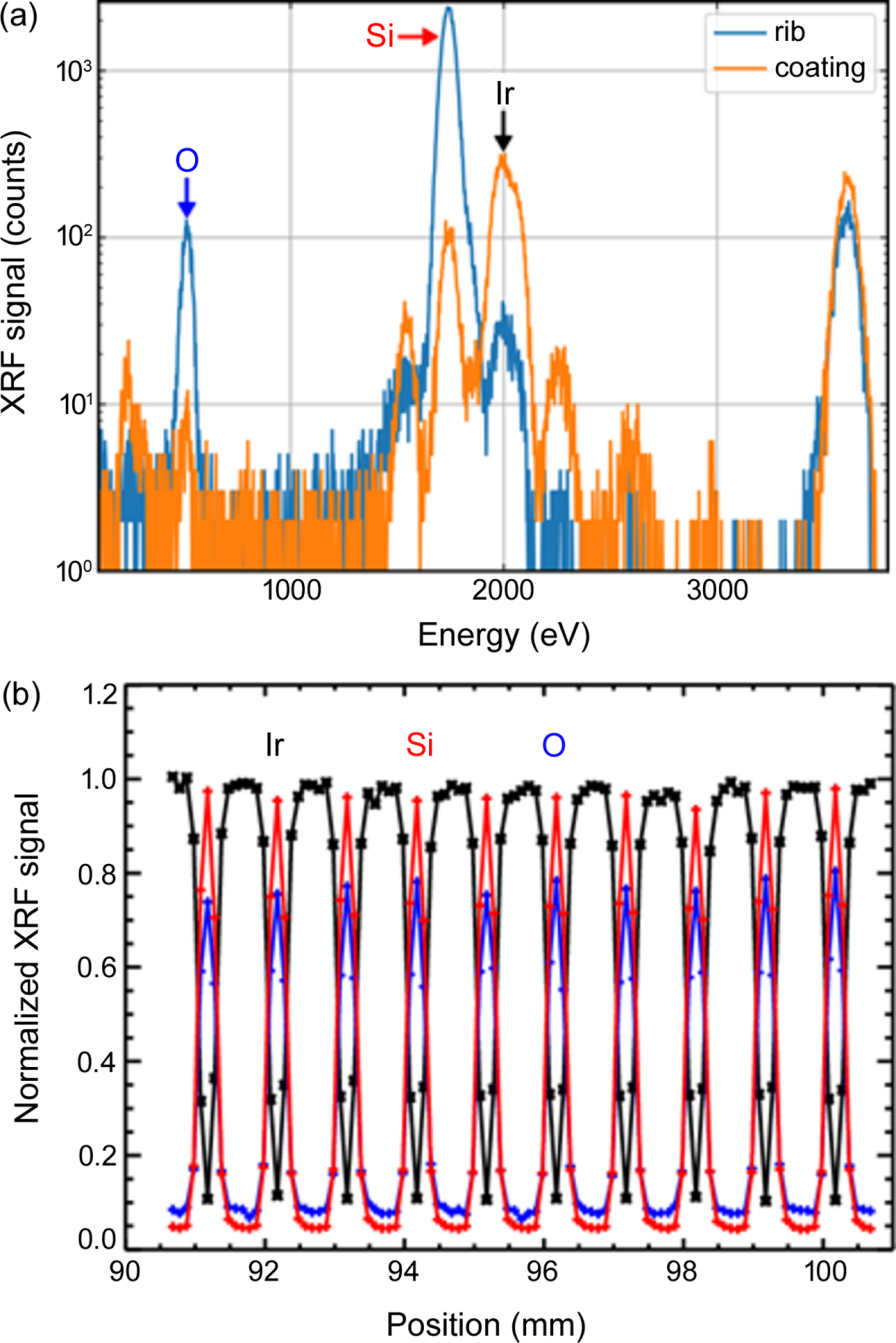
(*a*) XRF spectra obtained at an incident energy of 3.6 keV from an Ir-coated stripe and from an uncoated stripe above the ribs. (*b*) Normalized fluorescence intensities from a scan across the central region of the top surface.

A mirror module (MM-0061) was installed via the load lock in the UHV reflectometer as depicted in Fig. 5 ▸. This MM is a development sample, designed close to the geometry for the Athena middle row (row 8), and is composed of four mirror stacks, each with 34 reflecting plates. Every plate is 66 mm wide and 41 mm long, with a pore width of 0.8 mm and a rib width of 0.17 mm. The height of the pores is 0.6 mm, and the thickness of the silicon between the ribs is 0.17 mm. The in-vacuum goniometer head was used to align the ribs parallel to the beam, which had a size of about 0.2 mm × 0.2 mm. Prior to measuring the reflectance, an XRF scan was conducted on the top surface at an incidence angle of 0.814° and a photon energy of 3.6 keV. The results for the central region are shown in Fig. 6 ▸. As expected, alternating spectra are obtained for the coated stripes, dominated by Ir *M* fluorescence, and the uncoated stripes above the ribs, dominated by the Si *K* and O *K* fluorescence from the Si substrate which is covered by an SiO_2_ layer. The fact that the Ir fluorescence almost disappears above the narrow ribs proves that the beam is sufficiently small.

**Figure 7:**
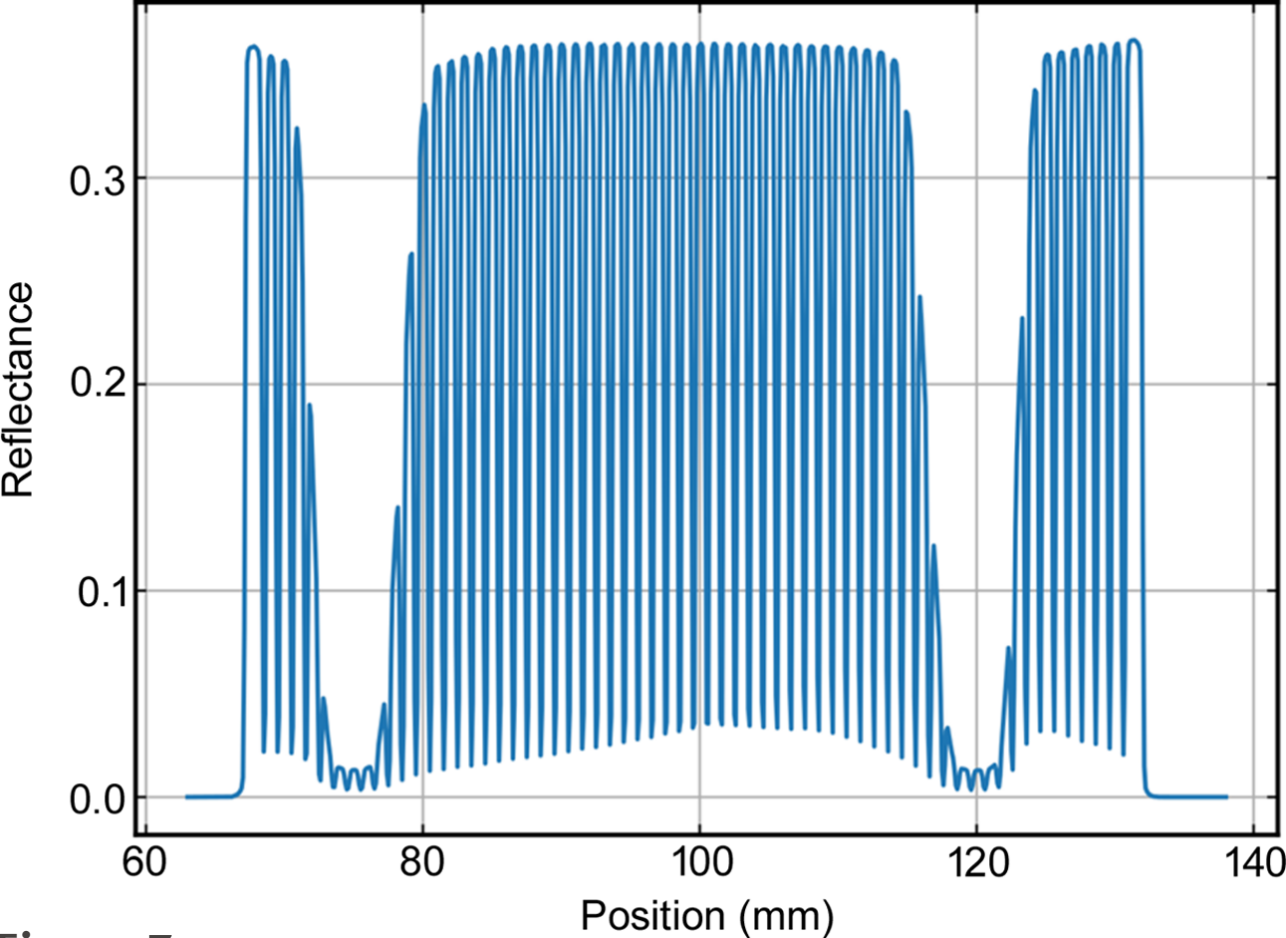
Scan over the total width of the MM in double reflection at 4 keV for a grazing incidence angle of 0.814°. The reflectance is about 35% on the Ir-coated stripes, and it vanishes when the beam is blocked by the ribs. The curvature of the stack, with a radius of about 0.7 m, caused the beam to be blocked by the reflecting plate around the 95 mm and 120 mm positions before it is reflected by the plate below.

The small beam was used to measure the reflectance through the pores of the MM. Plate 33 located below the top surface was measured at a grazing incidence angle of 0.814°. The GaP photodiode on the detector arm was centered at 3.256°, thus twice the kink angle between the first and the second stack. The horizontal translation range of the sample is large enough to scan the entire width of the MM, as shown in Fig. 7 ▸. Due to the curvature of the stack with a radius of approximately 0.7 m (as it is part of the large circular lens), the beam intercepts the reflecting plate at two positions while moving in a straight line. Beyond that, it is reflected by the plate below.

**Figure 8:**
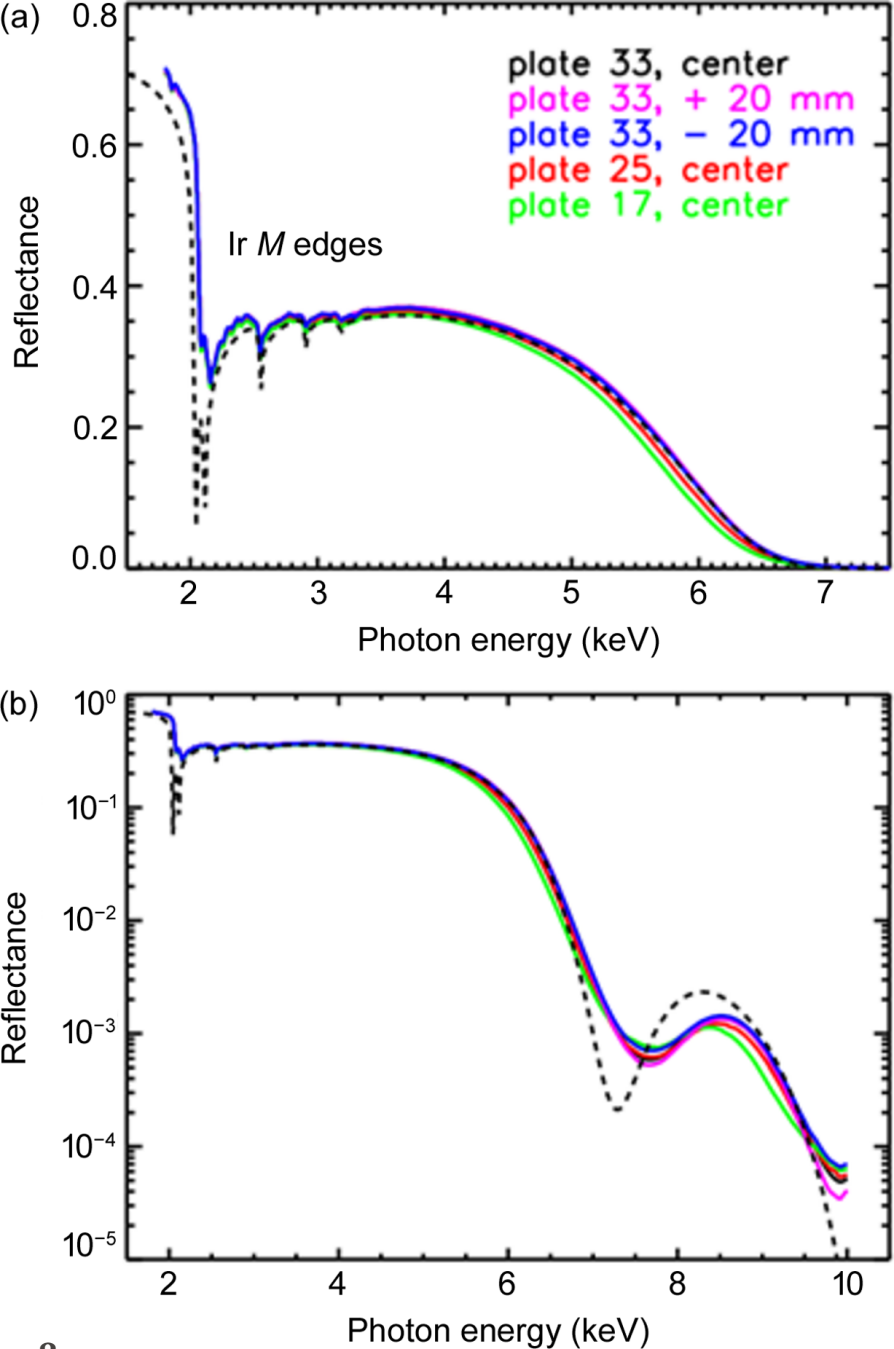
Energy-dependent reflectance (double reflection) at different positions (pores) of the MM. In the linear plot (*a*), the Ir *M* edges are clearly visible. As the incidence angle for plates 25 and 17 are slightly steeper (0.822° and 0.829° instead of 0.814° for plate 33), the reflectance decreases faster towards higher energies. From the position of the maximum and minimum in the logarithmic plot (*b*), an Ir layer thickness of about 10 nm can be confirmed. The calculated reflectance using the nominal values and optical constants from the literature (Henke *et al.*, 1993 ▸) is shown as a dashed line.

To measure the energy-dependent reflectance, the InSb crystals in the FCM were used up to 3.6 keV, while the Si crystals were employed for the higher photon energies up to 10 keV. Measurements were performed for three different pores (in the center and at ±20 mm) on plate 33, as well as in the center on plates 25 and 17, respectively. As shown in Fig. 8 ▸(*a*), the reflectance decreases from about 70% at 1.8 keV to about 35% at the Ir *M*_5_ edge. All other Ir *M* edges are also visible. Towards higher energies, the reflectance decreases even further, reaching 10% at approximately 6 keV. In this range, the effect of the steeper incidence angle of the other plates (0.822° for plate 25 and 0.829° for plate 17) is more pronounced. The vertical beam divergence is sufficiently small to observe this effect. Due to the geometry of the pores, it is not possible to perform X-ray reflectometry (XRR) with a Θ/2Θ scan to determine the thickness of the Ir layer. However, the thickness of about 10 nm can be confirmed from the positions of the minimum and maximum in the range between 7 keV and 9 keV in the logarithmic plot [Fig. 8 ▸(*b*)].

**Figure 9:**
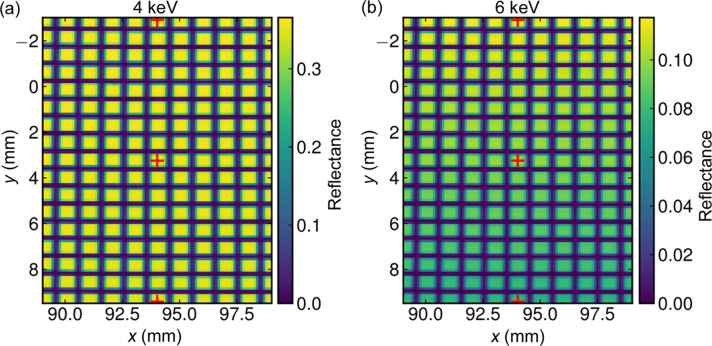
Raster scan of the reflectance (double reflection) at a fixed grazing incidence angle of 0.814° over the central area of 17 plates. The reflectance remains constant at (*a*) 4 keV, but it varies at (*b*) 6 keV due to the slightly steeper angle of the lower plates having more influence at higher photon energies. The measurement positions on plates 33, 25 and 17 for the energy scans in Fig. 8 ▸ are indicated.

The reflectance of each pore of the MM can be measured through a raster scan using the small beam. This would also be possible at the dedicated beamlines XPBF 1 and XPBF 2.0, but only at a fixed energy of 1 keV, while the FCM beamline allows for selection of any energy within the working range. Here, the entire width of the MM is also accessible in a single scan, but due to the limited vertical travel range of the goniometer head in the reflectometer the MM would have to be installed at different positions in the reflectometer to cover the full MM. Furthermore, the measurements with a step width of 0.1 mm are very time-consuming. Thus, only an area of 10 mm × 12.5 mm was scanned for Fig. 9 ▸. The pore structure is clearly visible and the reflectance is constant in the pores in the horizontal direction; however, in contrast to the measurement at 4 keV, it decreases at 6 keV in the vertical direction due to the increasing angle of the lower plates as result of the wedging.

**Figure 10:**
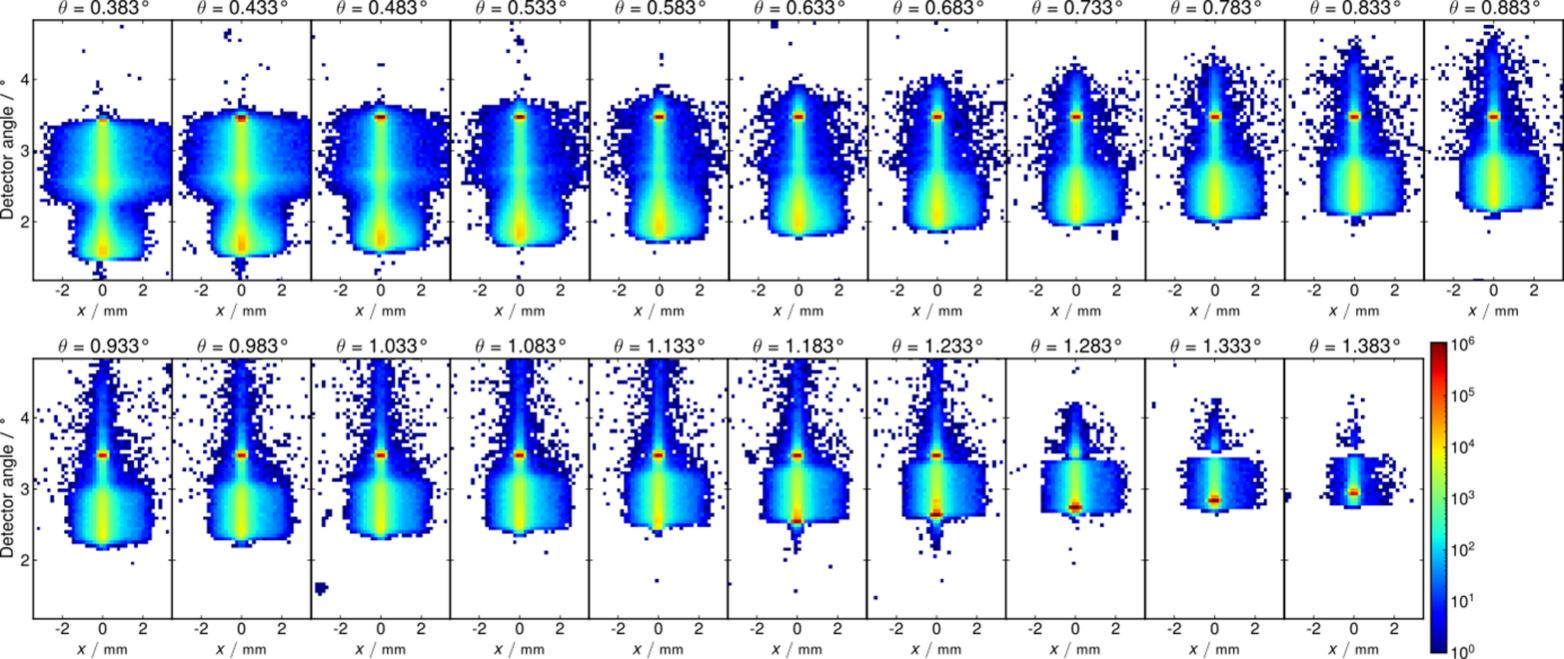
Images from the large-area detector taken for strong variations of the grazing incidence angle from about 0.4° to 1.4° on an (slightly different) MM. After double reflection, the beam remains fixed at a detector angle of about 3.4°. At steeper incidence angles above 1.18°, which are far away from the angle in the NewAthena optics, the beam can traverse the pores after a single reflection, and its position depends on the incidence angle.

As the optics for NewAthena are composed of about 500 MMs, the question of the required angular alignment accuracy of the MM with respect to each other might arise. Note that in the double-reflection configuration the total deviation is determined solely by the kink angle between the reflecting surfaces, which is about 0.8° (depending on the plate number) in the investigated MM. This is demonstrated in Fig. 10 ▸ for a very similar but not yet fully Ir-coated module (MM-0034) previously investigated at the FCM beamline. All images were obtained using the large-area hybrid pixel detector in the reflectometer, and the intensity is shown on a logarithmic scale. Despite varying the incidence angle in a large range from 0.4° to 1.4°, the specular reflected beam remains at a fixed position due to the double reflection. If the incidence angle is increased above 1.18°, the beam can pass through the pore after a single reflection. The beam position varies as expected with the incidence angle in this case. This condition can be met in a laboratory experiment where the angle of incidence can be significantly detuned and the detector is placed at a distance of 0.2 m. In contrast, in the X-ray observatory, the radiation sources are usually at an infinite distance and the detector is 12 m behind the optics.

## 4. Conclusions and outlook

In the PTB laboratory at BESSY II, the dedicated beamlines XPBF 1 and XPBF 2.0 are used to characterize and assemble MMs based on silicon pore optics for ESA’s NewAthena X-ray observatory. To ensure redundancy, a modified copy of XPBF 2.0, called MINERVA, has been installed at the ALBA synchrotron radiation facility in Spain (Heinis *et al.*, 2023 ▸). Two additional beamlines, similar to XPBF 2.0 and MINERVA, will be installed in the PTB laboratory for the mass production of MMs. This will allow us to begin the production of flight modules directly after the anticipated mission adoption in spring 2027. The launch of NewAthena is envisaged in 2037.

Meanwhile, the SPO production process will continue to be improved and the geometry of the MMs will be optimized for the different rows, including increased rib spacings of 2.4 mm and reduced silicon thickness between the ribs of 0.11 mm instead of 0.17 mm. The energy-dependent reflectance will be further optimized by applying a carbon overcoating on the Ir layer, which remains unaffected by the required cleaning procedures prior to stacking (Paredes-Sanz *et al.*, 2023 ▸). Reflectance measurements will be performed on such a module in 2024, covering a wide photon energy range from approximately 0.25 keV to 10 keV. The measurements will be conducted using the FCM beamline and a plane grating monochromator beamline in the PTB laboratory.

## Figures and Tables

**Table 1:** Parameters of the dedicated beamlines XPBF 1 and XPBF 2.0

	XPBF 1	XPBF 2.0
Photon energy (keV)	1.0	1.0
Monochromatization/collimation	2W/B_4_C flat multilayer mirrors and pinholes	W/Si multilayer coating on a toroidal mirror
Multilayer *d*-spacing (nm)	1.1	4.4
Multilayer Bragg angle (°)	15, vertical deflection	8.5, horizontal deflection
Beam size (with divergence <2 arcsec)	Typically 0.1 mm diameter	Up to 8 mm × 8 mm
Sample chamber diameter (mm)	600	700
Sample chamber height (mm)	780	1060
Sample chamber door diameter (mm)	400 (one door)	600 (two doors)
Horizontal sample translation (mm)	100	120
Vertical sample translation	150	150
Electronic autocollimators	2	2
Sample-to-detector distance (m)	5	12
Detector vertical translation (mm)	370	2100
Detector horizontal translation (mm)	60	165
Detector translation in the beam direction (mm)	0	1000
CCD pixel number	1300 × 1340	2048 × 2048
CCD pixel size (µm)	20	13.5
Laser tracker for detector	No	Yes
